# A Few Suggestions on Prescription Writing

**Published:** 1906-05

**Authors:** Luther A. Thomas

**Affiliations:** Buffalo, N. Y.


### A Few Suggestions on Prescription Writing.

By LUTHER A. THOMAS, Ph. G., Buffalo, N. Y.

THE proper use of vehicles means elegant pharmacy whether it be a formula made up by any of the manufacturing houses or in the form of a prescription written by the physician and compounded by the retail pharmacist. The pharmacist must compound our prescription as it is written; he may not alter the ingredients without our consent. So with us lies the responsibility if our prescription be a muddy or nauseating mixture which may bring about open rebellion on the part of the patient. Not infrequently have I had patients bring prescriptions back to the store, saying that they could not take the medicine, that the very sight and odor of the medicine produced nausea. In many cases the vehicle was at fault and in very few cases was the mixture prescribed for its psychological effect. The most the pharmacist can do under the circumstances is to refer the patient to the doctor. On one occasion a physician tried to cover up his ignorance by telling the patient that the prescription had been improperly compounded.

We will note that clear solutions having a distinctive color are much more agreeable to patients than muddy or even plain colorless mixtures. The manufacturing houses take advantage of this fact and the popularity of many of the proprietary pharmaceuticals lies in their elegant appearance and agreeable taste. One objection to this class of proprietary preparations is the high price that the manufacturer exacts for his pharmacal skill. Many of the fancy colored preparations on the market owe their color to cochineal, cudbear, various analine dyes, and allied coloring agents; while the vehicle is usually allied to the aromatic elixir of the U. S. P. I have no doubt that if the truth were known in each case, and we know it to be true of many, every ingredient of therapeutic value in these expensive and fancy named preparations is contained in the Pharmacopeia, while the so called "wonderful discovery" and "secret process" as advertised lies principally in the selection of a proper aromatic vehicle. It must be admitted that there are desirable combinations of drugs that do not give a clear solution with any vehicle, but this is the rare exception usually applying to vegetable preparations that might better be prescribed in solid form, using a solid or powdered extract of the drug, which always makes a good pill mass and can be enclosed in capsules.

Now let us consider a few of the common vehicles. First, let me emphasize that the soluble metallic salts should be given in solution, the reason for this being obvious. Potassium nitrate, for example, when taken into the stomach undiluted will not only produce irritation, but will actually erode and destroy the mucous membrane. For the metallic salts and other water soluble substances the aromatic waters may be employed. Of these the cinnamon and peppermint water are the two most commonly used. The cinnamon water of the U. S. P. should be diluted with equal parts of distilled water when used as a vehicle for metallic salts. The appearance of these solutions may be materially improved by the use of a small quantity of compound tincture of cardamon or compound tincture of lavender. A good adjuvant, diluent and vehicle for the various simple bitters is the infusion of compound gentian (gentian, bitter orange peel, and fresh lemon peel). In cases where a mild bitter that carries with it somewhat of a carminative effect is desired this infusion may be used alone. Even in the use of tr. nux vomica I believe it better to dilute so that the desired dose will be represented by one teaspoonful of the mixture, rather than have the patient use the medicine dropper, as this eliminates a possible chance for error in dosage.

Then we come to a class of preparations such as the tinctures and fluidextracts which are alcoholic or hydro-alcoholic and which will not give clear solutions with water. For such (and to be on the safe side we had better include most of our tinctures and fluid-extracts) there are two vehicles that I wish to speak of. The aromatic elixir of the U. S. P. is a dilute solution of the volatile oils of orange peel, lemon, anise, and coriander, with 37½ per cent of sugar and 25 per cent of alcohol. The presence of this quantity of alcohol aids materially in holding in solution the mixtures of the fluidextracts, but in dealing with tinctures and fluidextracts of distinctly resinous drugs from 10 per cent to 25 per cent of glycerine should be added. When we use tinctures or fluidextracts of a disagreeable taste, the doses of which approximate a teaspoonful, in order not to dilute to any great extent we may use a small quantity of the Aromatic Fluidextract of the U. S. P. which is alcoholic in nature and owes its aromatic properties to powdered cinnamon and powdered sugar, each 35 per cent, and powdered cardamon and powdered myristica, each 15 per cent. These same powders, when mixed in like proportion form the pulvis aromaticus of the U. S. P. which is a good diluent and aromatic for the various substances that are to be prescribed in powder form.

There is also another aromatic elixir which is found in the National Formulary that is a good vehicle and will disguise the bitter taste of drugs. I refer to the compound elixir taraxicum which is a clear brown solution containing several aromatics and mild stomachics, the principal ones being licorice and orange peel. This preparation contains about ten per cent of alcohol. It is a good vehicle for the various alkaloids of cinchona and other bitter substances.

Whenever possible it is better to use the aromatic waters or elixirs in preference to syrup, as saturated solutions of sugar are much more disturbing to the stomach. When a sweet preparation is desirable, as in some prescriptions for children, a small quantity of glycerine may be used, or saccharin, one grain to each ounce. The syrup of orange may be used and is very pleasant. Syrup of Tolu is a good dilutent and vehicle for the more potent medicated syrups and various expectorant mixtures. The syrup of sarsaparilla compound is the commonly used vehicle in the "mixed treatment" formula. As previously stated, however, the aromatic elixir is a better vehicle for that class of preparations which have as their therapeutic agents, the various alkaloids of opium, both natural and artificial, Terpen hydrate, and other substances whose solution is facilitated by the presence of alcohol.

The various fixed oils, such as cod liver oil and castor oil, the volatile oils such as eucalyptus and the various oily substances such as creosote, terebene, and guaiacol, should be dispensed in the form of an emulsion. We are familiar with acacia as an emulsifying agent. One part of the powdered acacia to five of the oil will yield a fairly permanent emulsion when properly prepared.

The yolk of egg is frequently used and it is usually considered that the yolks of two eggs will make 500 c.c. of a 50 per cent emulsion of cod liver oil. The extract of malt (U. S. P. 1905) is a good emulsifier and vehicle for this class of substances. It will emulsify its own weight of cod liver oil. These emulsions to be more palatable should be flavored. For this purpose various essential oils are used, such as oil of gaultheria, oil of sassafras, oil of coriander, and oil of bitter almonds. The combinations of the oils of gaultheria and sassafras each 15 minims to the pint is a good one. Oil of cloves, 10 minims to the pint, may be used.

For mixtures containing quinine and the allied substances licorice in some of its forms acts well in disguising the bitter taste. Compound elixir taraxicum, the syrup of licorice (Gregory) and the elixir adjuvans, U. S. P. 1905, are the more common preparations. When using the thick syrup of licorice the quinine or the bitter powder is gently stirred up with the syrup so that the particles are coated. The object of this would be defeated if the ingredients were trituated in a mortar.

As we know, the ordinary forms of iron cannot be combined with cinchona, gentian, and many other vegetable drugs, because of the inky mixtures formed. The National Formulary contains a tr. ferri citrochloride which is compatible with this class of vegetable substances. It is also known as the tasteless tincture of iron, and is practically of the same strength as the official tr. ferric chloride, differing in its alcoholic strength only. The use of the proprietary preparations of pepsin such as essence of pepsin, elixir of lactopeptine, etc. as vehicles where the digestive ferment is not particularly indicated is to be discouraged. It adds materially to the cost of the prescription and has no advantage over our ordinary pharmacopeal vehicles.

24 High Street.

